# Mangiferin attenuates lipopolysaccharide-induced neuronal injuries in primary cultured hippocampal neurons

**DOI:** 10.18632/aging.205830

**Published:** 2024-05-15

**Authors:** Hongling Tan, Dan Liang, Na Lu, Junli Zhang, Shiyan Zhang, Guojun Tan

**Affiliations:** 1 Department of Emergency Intensive Care Medicine, The Second Hospital of Hebei Medical University, Shijiazhuang 050000, China; 2 Department of Emergency Intensive Care Medicine, The First Hospital of Hebei Medical University, Shijiazhuang 050000, China; 3 Department of Medical, The Second Hospital of Hebei Medical University, Shijiazhuang 050000, China; 4 Department of Neurology, The Second Hospital of Hebei Medical University, Shijiazhuang 050000, China

**Keywords:** mangiferin, sepsis, sepsis-associated encephalopathy, lipopolysaccharide, neuron

## Abstract

Mangiferin, a naturally occurring potent glucosylxanthone, is mainly isolated from the *Mangifera indica* plant and shows potential pharmacological properties, including anti-bacterial, anti-inflammation, and antioxidant in sepsis-induced lung and kidney injury. However, there was a puzzle as to whether mangiferin had a protective effect on sepsis-associated encephalopathy. To answer this question, we established an *in vitro* cell model of sepsis-associated encephalopathy and investigated the neuroprotective effects of mangiferin in primary cultured hippocampal neurons challenged with lipopolysaccharide (LPS). Neurons treated with 20 μmol/L or 40 μmol/L mangiferin for 48 h can significantly reverse cell injuries induced by LPS treatment, including improved cell viability, decreased inflammatory cytokines secretion, relief of microtubule-associated light chain 3 expression levels and several autophagosomes, as well as attenuated cell apoptosis. Furthermore, mangiferin eliminated pathogenic proteins and elevated neuroprotective factors at both the mRNA and protein levels, showing strong neuroprotective effects of mangiferin, including anti-inflammatory, anti-autophagy, and anti-apoptotic effects on neurons *in vitro*.

## INTRODUCTION

Sepsis is a syndrome characterized by multiple organ dysfunction and a systemic inflammatory response caused by an infection that induces acute and long-term cognitive impairment [1]. Despite recent advances in anti-infection therapy and organ function support technology, the mortality rate from sepsis remains as high as 30–60% [2]. The central nervous system is particularly susceptible to the harmful effects of sepsis due to its high metabolic rate and blood-brain barrier (BBB) permeability [3]. Sepsis-associated encephalopathy (SAE) has various clinical manifestations, ranging from psychological dysfunctions such as anxiety, depression, and post-traumatic stress disorder to cognitive impairments such as memory decline and difficulty performing tasks, delirium, coma, or even death [4]. The pathophysiology of SAE has not been fully elucidated. Several commonly accepted mechanisms include impaired cerebral perfusion, altered neurotransmitters, impaired BBB, oxidative stress, inflammatory response, apoptosis, microglial activation, and metabolic disorders [5].

In addition to surgical administration, traditional Chinese medicinal compounds with antioxidant and anti-inflammatory effects have been considered potential therapeutic candidates for sepsis-induced organ dysfunction. Mangiferin (the C-glucopyranoside of 1,3,6,7-tetrahydroxyxanthone), a yellow polyphenolic natural product, has been widely studied for its pharmacological properties [6]. Mangiferin exhibits a range of pharmacological activities, including antioxidant, antiviral, immunomodulatory, anti-bacterial, anti-parasitic, anti-cancer, anti-diabetic, anti-asthmatic, hepatoprotective, anti-septic, anti-inflammatory activities, etc. [7–10]. Although mangiferin may not directly traverse the BBB, its secondary effect on brain metabolism [11] is effective in the treatment of neurodegenerative disorders [12] and cerebral ischemia-reperfusion injury [13]. In lipopolysaccharide (LPS)-induced sepsis-associated lung and kidney injuries, mangiferin also exhibits protective roles by downregulating vascular permeability and protecting against inflammatory and oxidative damage [7, 14, 15]. This comprehensive study focused on the effects of mangiferin on different signaling pathways, including phosphatidylinositol 3-kinase (PI3K)/protein kinase B (Akt), nuclear factor erythroid 2-like 2 (Nrf2)/eme oxygenase-1 (HO-1), and extracellular signal-regulated kinase1/2 (ERK1/2) [13]. However, the protective effects of mangiferin against LPS-induced neuronal damage and the underlying mechanisms have not yet been fully characterized. In this study, we investigated the protective effects of mangiferin against LPS-induced neuronal damage *in vitro* and explored the potential mechanisms underlying these effects. Mangiferin-treated neurons showed a significant reversal of LPS-induced cell injury, including improved cell viability and decreased inflammatory cytokine secretion. In addition, the anti-autophagic and anti-apoptotic effects of mangiferin reduced LC3 expression and the number of autophagosomes. Mangiferin eliminated pathogenic proteins and elevated neuroprotective factors at both mRNA and protein levels, showing a strong neuroprotective effect of mangiferin through anti-inflammatory, anti-autophagy, and anti-apoptotic effects on neurons *in vitro*.

## MATERIALS AND METHODS

### Drugs and antibodies

All drugs, kits, and antibodies used in this study were purchased as listed: mangiferin, Solarbio, China, IM0030; CCK-8 kit, Solarbio, CA1210; IL-1β ELISA kit, Multisciences, China, EK201B; IL-6 ELISA kit, Multisciences, EK2153; TNFα ELISA kit, Multisciences, EK282; Annexin V-FITC/PI kit, BioSharp, China, BL110A; anti-LC3, Proteintech, China, 14600-1-AP; anti-Aβ42, Bioss, USA, BS0107R; Anti-p-Tau, Affinity, USA, AF3148; Anti-APJ, Affinity, DF13350; Anti-VEGF, Affinity, AF5131; Anti-S100β, Affinity, DF6116; Anti-NSE, Affinity, AF5473.

### Primary hippocampal neuron culture

Neonatal C57BL/6 mice, male, aged 1–2 days and weighing 1 g, were purchased from Jinan Pengyue Experimental Animal Breeding Co., Ltd. (China). Primary cultured hippocampal neurons were prepared from newborn mice of either sex within 24 h after the experiment. Briefly, the hippocampus was dissociated and mechanically and chemically triturated until a single-cell suspension was obtained. The cells collected were seeded in 77% DMEM/F12 basal culture medium supplied with 20% FBS, 2% B27, and 1% P/S complete medium in a humid atmosphere with 5% CO_2_ at 37°C.

### CCK-8 assay

The neurons were seeded in 96-well plates and kept in DIV12. Cells were incubated with 5 μmol/L, 10 μmol/L, 20 μmol/L, 40 μmol/L, and 60 μmol/L of mangiferin for 24 h, then 10 μL/well of reaction buffer was added. After 3 h of incubation, the absorbance was measured at 450 nm using a microplate reader. To establish an *in vitro* cell damage model of LPS-induced SAE, cells were exposed to 1 μg/ml of LPS and then subjected to a CCK-8 assay.

### ELISA

The neurons were exposed to 1 μg/mL LPS and then incubated with 20 μmol/L or 40 μmol/L mangiferin for 48 h. The supernatants were collected and the concentration of IL-1β, IL-6, and TNF-α was measured by ELISA assay according to the manufacturer’s protocol.

### Flow cytometry

Annexin V-FITC and PI staining (Biosharp, BL110A) combined with flow cytometry were performed to assess cell apoptosis. Briefly, cells were harvested by centrifugation at 1000 rpm for 5 min at 4°C after trypsin digestion. After washing with PBS, cells were resuspended with 200 μL binding buffer and incubated with 5 μL Annexin-FITC and 5 μL PI at room temperature for 10 min. Finally, cells were classified using a BD Accuri C6 flow cytometer (excitation = 488 nm; emission = 530 nm).

### Immunofluorescence

The neurons in DIV12 were washed once with CytoBuffer cells (50 μmol/L MES (pH 6.1), 5 μmol/L MgCl_2_, 3 μmol/L EGTA, and 5 μmol/L glucose) and then fixed in freshly prepared 4% paraformaldehyde for 40 min at room temperature. To quench autofluorescence, cells were washed twice for 10 min each with 1 mg/mL NaBH_4_ in TBS (20 μmol/L Tris pH 7.5, 154 μmol/L NaCl, 2 μmol/L EGTA, and 2 μmol/L MgCl_2_). Cells were then permeabilized and blocked with TBS containing 2% bovine serum albumin and 0.02% saponin (0.5 mL/well) for 1.5 h. After incubation with primary antibodies overnight at 4°C, the cells were washed three times with TBS and then incubated for 1 h with secondary antibodies. After three washes with TBS, cells were mounted on slides with a mounting medium containing 50% DAPI. Cells were scanned using an inverted fluorescence microscope Olympus (IX73).

### Transmission electron microscope

Cultured cells from different treatment groups were fixed with 2.5% glutaraldehyde for 4 h, followed by secondary fixation with 1% osmium tetroxide for 2 h. The cells were then dehydrated (30%, 50%, 70%, 80%, 85%, 90%, 100% alcohol gradients), infiltrated and embedded with acetone: resin (2:1), acetone: resin (1:1), resin step by step for 8 h in a 37°C incubator. After the samples were polymerized, ultrathin serial 80-nm sections were cut with a Diatome diamond knife using a Leica UC6 ultramicrotome. The sections collected on the grids were stained with 1% uranyl acetate (wt/vol) followed by lead citrate and finally examined and photographed using a transmission electron microscope.

### Western blot

For Western blot, the samples were separated on a 10% SDS-PAGE gel and transferred to a nitrocellulose filter membrane. The membrane was blocked for 1 h with 5% non-fat dried milk (w/v) prepared in PBST (in μmol/L: 135 NaCl, 2.7 KCl, 1.5 KH_2_PO_4_, 8 K_2_HPO_4_, pH 7.2, 0.05% (v/v) Tween 20). After washing with PBST, the membrane was incubated with the primary antibody in PBST containing 2% bovine serum albumin at 4°C overnight. After three washes with PBST, the secondary antibody was applied at room temperature for 1 h. After three washes with PBST, the membranes were scanned using an Odyssey infrared imaging system (LI-COR Biosciences, USA). Aβ42 (Bioss, BS0107R, 1:1000); p-Tau (Affinity, AF3148, 1:1000); S100β (Affinity, DF6116, 1:1000); NSE (Affinity, AF5473, 1:1000); APJ (Affinity, DF13350, 1:1000); VEGF (Affinity, AF5131, 1:1000); GAPDH (Affinity, AF7021, 1:5000). Horseradish peroxidase labeled goat anti-rabbit IgG (H + L) (Beyotime Biotech Inc., China, A0208, 1:10000); Horseradish peroxidase labeled goat anti-mouse IgG (H + L) (Beyotime Biotech Inc., A0216, 1:10000).

### RNA extraction and qRT-PCR

RNA extraction and qRT-PCR were performed as previously described. Briefly, total RNA was extracted using TRIZOL (Solarbio, R1100) and chloroform. RNA concentration was measured using a NanoDrop 2000c Spectrophotometer (Thermo Fisher Scientific, Rockford, IL, USA). The cDNA was reverse transcribed from total RNA according to the manufacturer’s instructions. Real-time quantitative PCR experiments were performed on a Quant Studio PCR system (Applied Biosystems, Foster City, CA, USA) using SYBR qPCR Master mix (Tolobio, China, 22204-01) with specific gene primers (Table 1). The qRT-PCR reactions were performed as follows: 1 cycle of 95°C for 3 min, 40 cycles of 95°C for 30 s, and 55°C for 20 s. Melting analysis was used to confirm the specificity of the amplicons. Gene expression levels were normalized to GAPDH mRNA levels using the comparative CT method. Three independent experiments were performed in triplicate for each sample.

**Table 1:** Primer sequences used in this study.

**Primer**	**Sequence**
Mus GAPDH-F	5′-GCCTCCTCCAATTCAACCCT-3′
Mus GAPDH-R	5′-CTCGTGGTTCACACCCATCA-3′
Mus Aβ42-F	5′-CCCAAGATCCTGATAAACTTCCCAC-3′
Mus Aβ42-R	5′-AGGCTCGACTTCATTTTCGGT-3′
Mus p-Tau-F	5′-CCACACGGAGATCCCAGAAG-3′
Mus p-Tau-R	5′-GTGTTGGTAGGGATGGGGTG-3′
Mus APJ-F	5′-CCCTTCCCCTCAAACCTTCC-3′
Mus APJ-R	5′-CAGCCTTAGGACCAGATGCC-3′
Mus VEGF-F	5′-GGGAGTCTGTGCTCTGGGAT-3′
Mus VEGF-R	5′-GGTGTCTGTCTGTCTGTCCG-3′
Mus s100β-F	5′-CTGATCGCCTACACCCTTCC-3′
Mus s100β-R	5′-CACAGTCCTCGACTCTCAGC-3′
Mus NSE-F	5′-GTCGGCATCCAGATAGTGGG-3′
Mus NSE-R	5′-AAGGGGATCACAGCACACTG-3′

### Experimental design and statistical analysis

All data from the experiments are presented as mean ± SD and statistical analysis was performed using GraphPad Prism software. Statistical comparisons were performed using the two-tailed unpaired *t*-test, one-way ANOVA with Dunnett’s analysis, or two-way ANOVA with Sidak analysis. The Sidak’s post-hoc test was used. Statistical significance was set at *P* < 0.05 was considered significant.

### Availability of data and materials

The datasets used and/or analyzed in the current study are available from the corresponding author upon reasonable request.

## RESULTS

### Primary cultured neuron preparation

**Figure 1:**
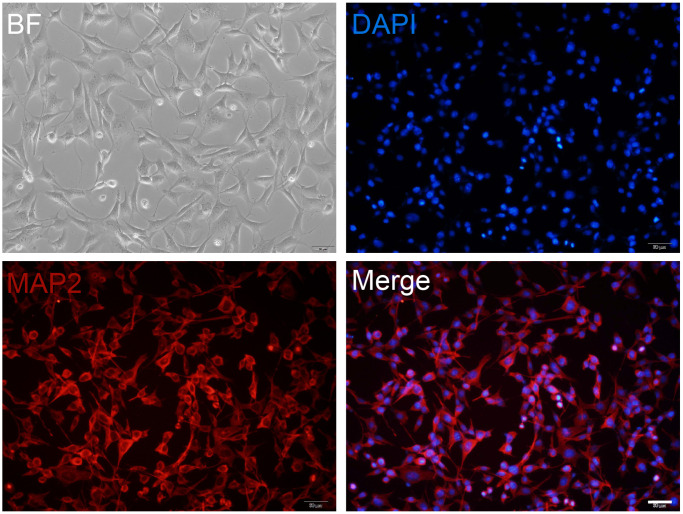
**Primary cultured neuron preparation.** Primary cultured hippocampal neurons are maintained at DIV12 and are observed in a bright field (BF) or stained with MAP2 (red). DAPI (blue) is used as a nuclear marker. Scale bar, 50 μm.

To obtain high purity and healthy neurons for screening the effective concentration of mangiferin, we examined cultured cells using MAP2 antibody staining. The images in Figure 1 showed almost all the cells were labeled with MAP2 (Figure 1), indicating the high purity of the hippocampal neurons.

### Screening of optimal concentration of mangiferin for cell protection study

**Figure 2:**
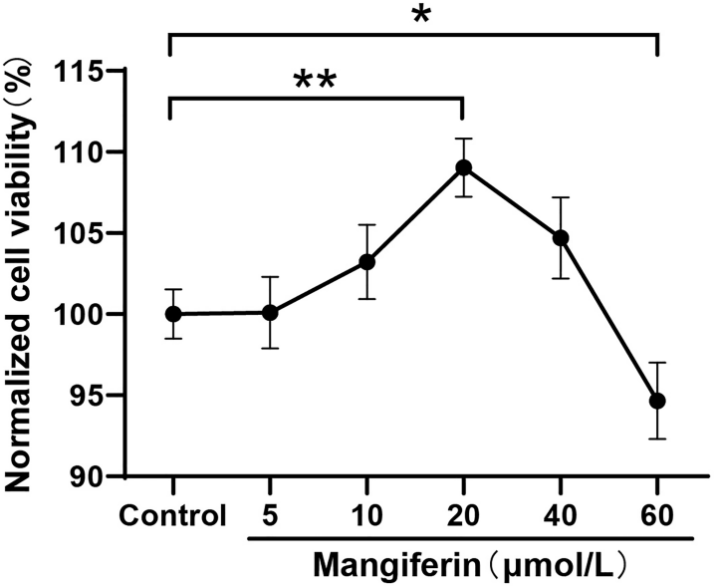
**Neuron viability with different concentrations of mangiferin treatment.** DIV12 neurons are incubated with different doses of mangiferin for 24 h and absorption is measured at 450 nm using a microplate reader. Data are collected from three independent experiments and presented as mean ± SD. One-way ANOVA is used for statistical analysis; ^*^P < 0.05, ^**^P < 0.01, ^***^P < 0.001.

To explore the optimal concentration of the protective effectiveness of mangiferin, we checked neuron viability after 24 h of treatment with different doses of mangiferin (5 μmol/L, 10 μmol/L, 20 μmol/L, 40 μmol/L, and 60 μmol/L) by Cell Counting Kit 8 (CCK-8). The results showed that 20 μmol/L and 40 μmol/L of mangiferin increased cell viability to 109.0% and 104.7%, respectively (Figure 2, one-way ANOVA, F = 15.65, ^***^P < 0.001) compared to the control group, while 60 μmol/L of mangiferin showed mild cytotoxicity and increased cell viability to 94.7% (^*^P = 0.038). No significant changes in cell viability were observed in the other groups.

### Mangiferin improves cell viability under LPS-exposed conditions

**Figure 3:**
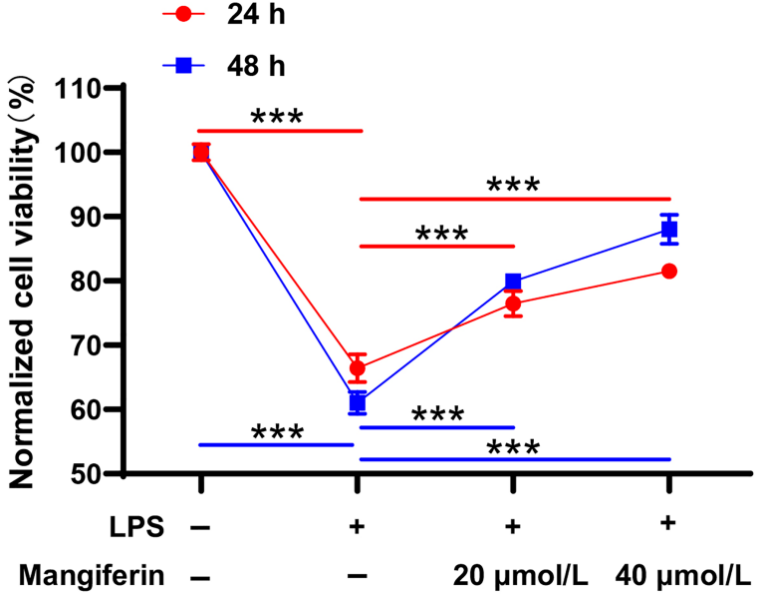
**Mangiferin protects neurons from LPS injury.** LPS-exposed neurons are incubated with 20 μmol/L or 40 μmol/L of mangiferin for 24 h or 48 h and then analyzed with the CCK8 assay. LPS treatment significantly affected cell viability (66.41% in 24 h, 61.07% in 48 h, ^***^P < 0.001 compared to the PBS control group), while 20 μmol/L (76.50% in 24 h, 79.91% in 48 h, ^***^P < 0.001) and 40 μmol/L (81.54% in 24 h, 88.02% in 48 h, ^***^P < 0.001) of mangiferin countered the LPS-induced phenotype. Data are presented as mean ± SD. One-way ANOVA is used for statistical analysis; ^***^P < 0.001.

Lipopolysaccharides (LPS), components of the outer membrane of Gram-negative bacteria, are key activators of immune responses during SAE [16]. To test the protective effectiveness of mangiferin, we used 20 μmol/L and 40 μmol/L of mangiferin to counteract neuron injuries induced by 1 μg/mL LPS. With 24 h or 48 h of mangiferin incubation, the CCK-8 assay data showed that LPS significantly damaged cell viability (Figure 3, 66.41% in 24 h, 61.07% in 48 h, ^***^P < 0.001 compared to the PBS control group); however, mangiferin reversed damage at 20 μmol/L (Figure 3, 76.50% in 24 h, 79.91% in 48 h, ^***^P < 0.001) and 40 μmol/L (Figure 3, 81.54% in 24 h, 88.02% in 48 h, ^***^P < 0.001). These data strongly supported the idea that 20 μmol/L or 40 μmol/L mangiferin protected neurons from LPS injury.

### Mangiferin shows an anti-inflammatory effect on LPS-exposed cells

**Figure 4:**
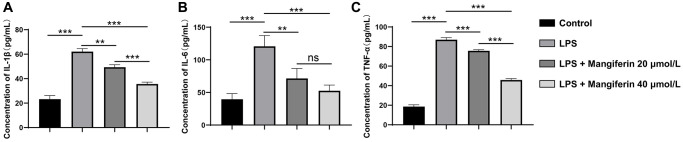
**Anti-inflammatory effect of mangiferin on LPS-exposed cells.** The concentrations of IL-1β, IL-6, and TNF-a in mangiferin-treated culture medium are measured by ELISA assay. Data are presented as mean ± SD. One-way ANOVA is used for statistical analysis. (**A**) Concentrations of IL-1β in control, LPS, 20 μmol/L mangiferin with LPS, and 40 μmol/L mangiferin with LPS are 23.23 pg/mL, 62.11 pg/mL, 49.37 pg/mL, and 35.64 pg/mL, respectively, all ^***^P < 0.001. (**B**) Concentrations of IL-6 in control, LPS, 20 μmol/L mangiferin with LPS, 40 μmol/L mangiferin with LPS are 39.52 pg/ml, 120.70 pg/mL, 71.41 pg/mL, and 52.57 pg/mL, respectively. ^**^P = 0.0066 for LPS vs. 20 μmol/L mangiferin with LPS, *P* = 0.23 for 20 μmol/L mangiferin with LPS vs. 40 μmol/L mangiferin with LPS, no significance is observed. All other comparisons are presented as ^***^P < 0.001. (**C**) Concentrations of TNF-a in control, LPS, 20 μmol/L mangiferin with LPS, and 40 μmol/L mangiferin with LPS are 18.61 pg/mL, 86.98 pg/mL, 75.60 pg/mL, and 45.80 pg/mL, respectively, all ^***^P < 0.001.

LPS treatment induces inflammation, autophagy, and apoptosis [16]. To assess the anti-inflammatory effect of mangiferin, we detected the secretion of inflammatory cytokines after 48 h of 20 μmol/L or 40 μmol/L of mangiferin treatment on LPS-exposed neurons using the ELISA assay. LPS markedly elevated the levels of IL-1β, IL-6, and TNF-a; however, 20 μmol/L and 40 μmol/L of mangiferin markedly alleviated the increase in LPS-induced proinflammatory factor levels (Figure 4, one-way ANOVA for IL-1β, IL-6, and TNF-a analysis; F = 160.8, ^***^P < 0.001 for IL-1β; F = 23.91, ^***^P = 0.003 for IL-6; F = 980.7, ^***^P < 0.001 for TNF-a). These results indicated that mangiferin improved the inflammatory response.

### Mangiferin treatment inhibits the autophagy process induced by LPS

**Figure 5:**
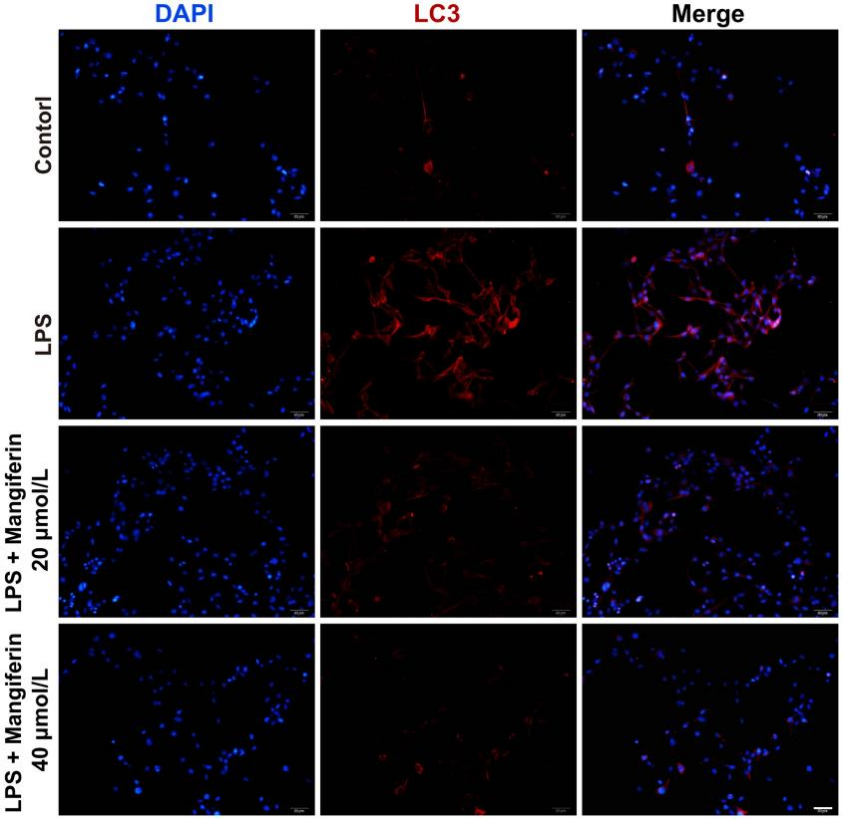
**Mangiferin treatment inhibits the autophagy process induced by LPS.** Neurons are stained with LC3 (red) to label the autophagosomes and DAPI (blue) to mark the nucleus. Scale bars, 50 μm.

**Figure 6:**
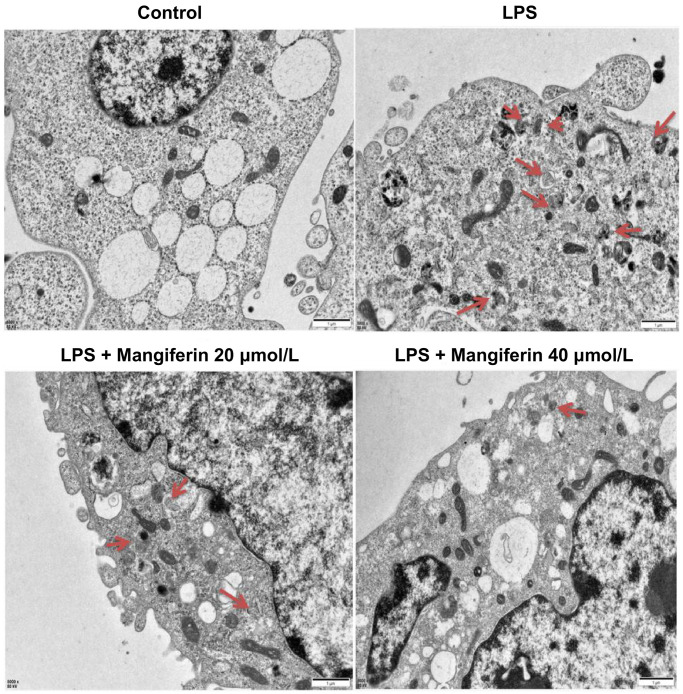
**Mangiferin treatment alleviates the number of autophagosomes induced by LPS by TEM.** Different neuronal treatments were performed for transmission electron microscopy (TEM). Red arrowheads indicate autophagosomes. Scale bars, 1 μm.

Apoptosis is always accompanied by autophagy, and LC3 is a widely accepted molecular marker of autophagy activation. To verify the role that mangiferin played during autophagy, we examined LC3 expression after 48 h of 20 μmol/L or 40 μmol/L of mangiferin treatment on LPS-exposed neurons by immunofluorescence. Both LC3 positive cells and the fluorescence intensity of LC3 increased substantially (Figure 5), and 20 μmol/L and 40 μmol/L of mangiferin reversed these phenotypes, indicating that mangiferin inhibited the LPS-induced autophagy process. To directly examine the anti-autophagic effect of mangiferin, we used a transmission electron microscope (TEM) to examine the ultrastructure of autophagosomes. Autophagosomes usually have a vacuolar-like structure of bilayer or multilayer membranes that contain cytoplasmic components such as mitochondria, endoplasmic reticulum, and ribosomes [17]. Consistent with the data of LC3 fluorescence, the number of autophagosomes increased markedly in the LPS group, while 20 μmol/L or 40 μmol/L of mangiferin alleviated the number of autophagosomes (Figure 6). These data demonstrate the anti-autophagic effect of mangiferin.

### Mangiferin shows an anti-apoptotic effect on LPS-exposed cells

**Figure 7:**
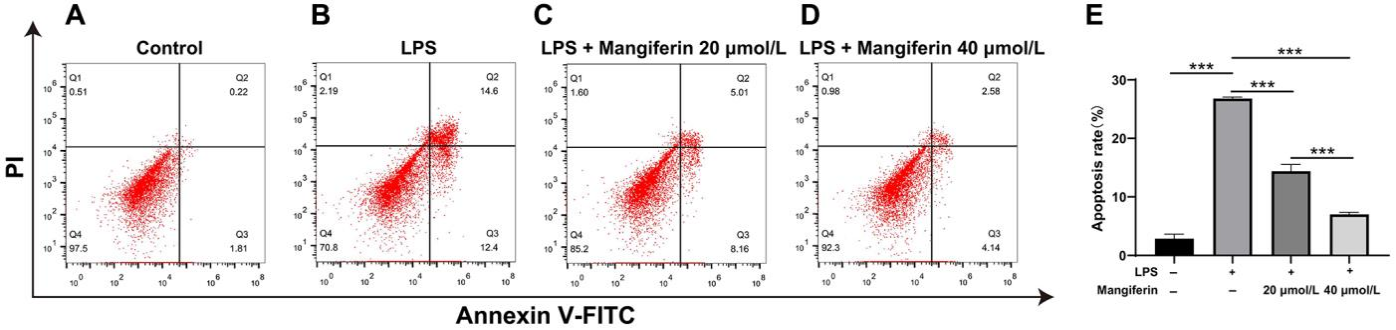
**Mangiferin mediates an anti-apoptotic effect in LPS-exposed cells.** (**A**–**D**) The plots show the flow cytometry of different treatments, and the apoptosis rates are analyzed (**E**). In the plots, quadrant Q4 shows surviving cells, Q2 and Q3 show dead cells, and Q1 shows cell debris; Q2 and Q3 are used to assess apoptosis. The apoptosis rates in the control, LPS, 20 μmol/L mangiferin with LPS, 40 μmol/L mangiferin with LPS are 2.87%, 26.8%, 14.39%, and 7.02% respectively, all ^***^P < 0.001.

Autophagy is normally protective of cell survival, but excess autophagy leads to apoptosis [18]. To assess the anti-apoptotic effect of mangiferin, we detected cell apoptosis after 48 h of 20 μmol/L or 40 μmol/L of mangiferin treatment on LPS-exposed neurons by flow cytometry. As shown in Figure 7, LPS exposure significantly increased the apoptosis rate of neurons (Figure 7A, 7B), while 20 μmol/L and 40 μmol/L of mangiferin reversed LPS-induced neurons apoptosis (Figure 7C, 7D, one-way ANOVA, F = 636.8, ^***^P < 0.001), showing an anti-apoptotic effect of mangiferin (Figure 7E).

### Mangiferin eliminates pathogenic proteins and increases neuroprotective factors in LPS-challenged cells

**Figure 8:**
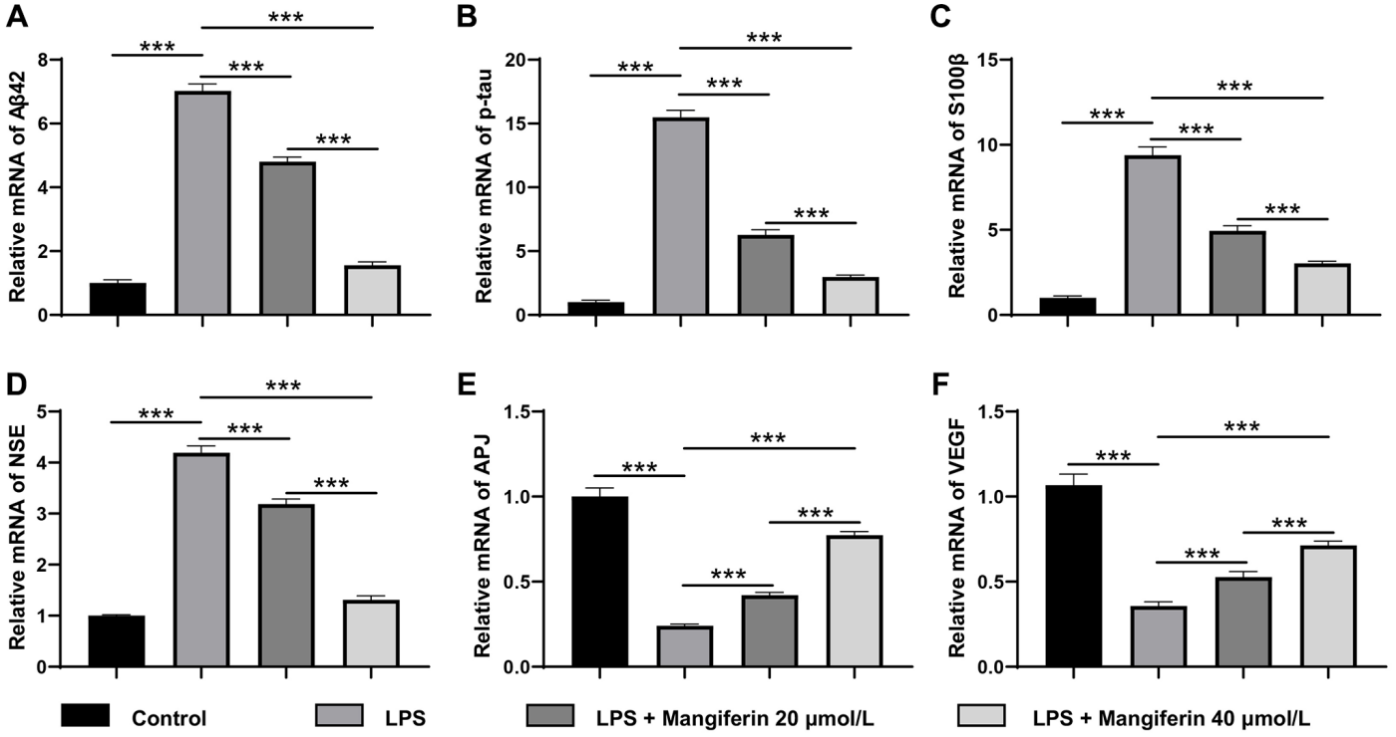
**Mangiferin eliminates pathogenic proteins and increases neuroprotective factors in LPS-challenged cells by RT-PCR.** (**A**) The relative mRNA levels of Aβ42 in LPS, 20 μmol/L mangiferin with LPS, and 40 μmol/L mangiferin with LPS compared to control are 7.02, 4.80, and 1.56 times, respectively, all ^***^P < 0.001. One-way ANOVA, F = 1199, ^***^P < 0.001. (**B**) Relative mRNA levels of p-tau in LPS, 20 μmol/L mangiferin with LPS, and 40 μmol/L mangiferin with LPS compared to control are 15.49, 6.27, and 3.00 times, respectively, all ^***^P < 0.001. One-way ANOVA, F = 1013, ^***^P < 0.001. (**C**) Relative mRNA levels of S100β in LPS, 20 μmol/L mangiferin with LPS, and 40 μM mangiferin with LPS compared to the control are 9.39, 4.95, and 3.03, respectively, all ^***^P < 0.001. One-way ANOVA, F = 446, ^***^P < 0.001. (**D**) Relative mRNA levels of NSE in LPS, 20 μmol/L mangiferin with LPS, 40 μmol/L mangiferin with LPS compared to control were 4.19, 3.19, and 1.31 times, respectively, all ^***^P < 0.001. One-way ANOVA, F = 834, ^***^P < 0.001. (**E**) Relative mRNA levels of APJ in LPS, 20 μmol/L mangiferin with LPS, and 40 μmol/L mangiferin with LPS compared to control are 0.24, 0.42, and 0.77 times, respectively, all ^***^P < 0.001. One-way ANOVA, F = 1688, ^***^P < 0.001. (**F**) Relative mRNA levels of VEGF in LPS, 20 μmol/L mangiferin with LPS, and 40 μmol/L mangiferin with LPS compared to control are 0.36, 0.53, and 0.71 times, respectively, all ^***^P < 0.001. One-way ANOVA, F = 396, ^***^P < 0.001.

**Figure 9:**
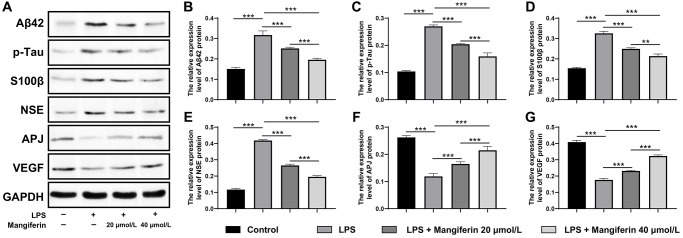
**Mangiferin eliminates pathogenic proteins and elevates neuroprotective factors in LPS-challenged cells by Western blot.** (**A**) Western blot showed the protein level of Aβ42, p-tau, S100β, NSE, APJ, and VEGF change with different treatments. (**B**–**G**) Quantitative analysis of A, protein levels of Aβ42 in LPS, 20 μmol/L mangiferin with LPS, 40 μmol/L mangiferin with LPS compared to control were 2.10, 1.67, and 1.30 times, respectively, all ^***^P < 0.001. One-way ANOVA, F = 111.7, ^***^P < 0.001. Protein levels of p-tau in LPS, 20 μmol/L mangiferin with LPS, 40 μmol/L mangiferin with LPS compared to the control are 2.60, 1.94, and 1.53 times, respectively, all ^***^P < 0.001. One-way ANOVA, F = 285.7, ^***^P < 0.001. Protein levels of S100β in LPS, 20 μmol/L mangiferin with LPS, and 40 μmol/L mangiferin with LPS compared to the control are 2.11, 1.61, and 1.38 times, respectively, ^**^P = 0.0026 for 20 μmol/L mangiferin with LPS vs. 40 μmol/L mangiferin with LPS. One-way ANOVA, F = 241.0, ^***^P < 0.001. Protein levels of NSE in LPS, 20 μmol/L mangiferin with LPS, 40 μM mangiferin with LPS compared to the control are 3.57, 2.27, and 1.67 times, respectively, all ^***^P < 0.001. One-way ANOVA, F = 990.9, ^***^P < 0.001. Protein levels of APJ in LPS, 20 μmol/L mangiferin with LPS, 40 μmol/L mangiferin with LPS compared to control are 0.45, 0.63, and 0.82 in fold, respectively, ^**^P = 0.0028 for LPS vs. 20 μmol/L mangiferin with LPS, ^**^P = 0.0015 for 20 μmol/L mangiferin with LPS vs. 40 μmol/L mangiferin with LPS. One-way ANOVA, F = 108.3, ^***^P < 0.001. Protein levels of VEGF in LPS, 20 μmol/L mangiferin with LPS, 40 μM mangiferin with LPS compared to control were 0.43, 0.57, and 0.79 times, respectively, all ^***^P < 0.001. One-way ANOVA, F = 467.7, ^***^P < 0.001.

Mangiferin has also been reported to be a multi-potent natural product preventing neurodegeneration and sepsis [19]. To detect whether mangiferin could eliminate pathogenic proteins induced by LPS, we observed the mRNA and protein expression of amyloid-β (Aβ42), p-tau, S100β, and neuron-specific enolase (NSE), which are usually adopted to assess the damage to SAE. As shown in Figures 8A–8D and 9A–9E, LPS treatment significantly increased mRNA and protein levels of Aβ42 and p-tau, S100β, and NSE. Furthermore, the mRNA and protein levels of the cardiovascular system protector apelin-angiotensin receptor-like 1 (APJ, Figures 8E, 9F) and the cell angiogenesis promoter vascular endothelial growth factor A (VEGF, Figures 8F, 9G) decreased significantly, indicating that the cells were severely damaged by LPS. In agreement with the above results, both 20 μmol/L and 40 μmol/L of mangiferin decreased the mRNA and protein level of pathogenic proteins (Aβ42 and p-tau, S100β, and NSE) and increased the mRNA and protein levels of neuroprotective factors (APJ, VEGF), representing the powerful protective effects of mangiferin.

## DISCUSSION

Intraperitoneal (i.p.) injection of lipopolysaccharide (LPS) is a widely accepted method for establishing animal models of sepsis. Increasing *in vivo* studies have shown that LPS-induced inflammation causes inappropriate activation of microglia and astrocytes, damages the BBB, and negatively affects mitochondrial function, oxidative/nitrative stress, and apoptosis [20, 21]. Although mangiferin shows excellent protective effects against sepsis-induced lung or kidney injury [14, 15], the neuroprotective effects of mangiferin have not been investigated in LPS-induced sepsis using primary cultured hippocampal neurons. In this study, we screened for the optimal concentration of mangiferin using a CCK-8 assay. The CCK-8 assay was used to investigate the toxicity of mangiferin (5–60 μmol/L) to neurons; 20 μmol/L and 40 μmol/L mangiferin increased neuron viability, while the higher concentration of mangiferin showed neurotoxicity (Figure 2). Therefore, we applied 20 μmol/L and 40 μmol/L of mangiferin for subsequent experiments. In this LPS-induced neuron damage model, 20 μmol/L and 40 μmol/L of mangiferin not only increased neuron viability (Figure 3), but also markedly alleviated the secretion of inflammatory cytokines (IL-1β, IL-6, and TNF-a) (Figure 4). These data are consistent with previous results showing that mangiferin has anti-inflammatory effects on LPS-induced inflammatory responses in macrophages [22] and microglia [23], indicating a similar anti-inflammatory effect of mangiferin on LPS-induced neuronal damage.

Autophagy is a conserved degradation process that maintains cellular homeostasis and the clearance of damaged organelles [24] and is always accompanied by cell apoptosis, which is a complex process that can promote cell survival or cell death [22]. In non-neuronal cells, autophagy and apoptosis have been shown to be interconnected by several molecular nodes of crosstalk, enabling the coordinated regulation of cell survival during mild stimulation of autophagy or cell death during excess autophagy [25]. Autophagosome formation is usually monitored by measuring the processing of LC3, a cytosolic protein that, in autophagy induction, is converted from LC3-I to LC3-II by lipid conjugation [26]. Immunofluorescence analysis showed that mangiferin inhibited LPS-induced LC3 expression (Figure 5). Meanwhile, the number of autophagosomes decreased in LPS-treated neurons following administration of mangiferin (Figure 6). However, mangiferin treatment reversed this phenotype, indicating that mangiferin inhibited LPS-induced overactivation of autophagy. Furthermore, flow cytometry showed that mangiferin treatment reversed LPS-induced neuronal apoptosis (Figure 7), suggesting that mangiferin effectively protects neurons from LPS-induced apoptosis. Combined with this part of the work, mangiferin eased neurons from stimulation of LPS, thus protecting neurons from overactivated autophagy and subsequent cell apoptosis.

Extracellular amyloid-β plaques and intracellular tau-containing neurofibrillary tangles were considered a biomarker of Alzheimer’s disease [27]. *In vivo* experiments showed that even a single i.p. injection of LPS led to intracellular accumulation of Aβ42 in hippocampal pyramidal neurons with unknown precise mechanisms [28]. In this LPS-exposed neuron, protein and mRNA levels of these two pathogenic proteins (Aβ42 and p-tau) increased, as well as two other brain injury biomarkers (S100β and NSE) used mainly in brain trauma, cerebral stroke, SAE, and hypoxic ischemia encephalopathy [29, 30]. Mangiferin not only counteracted the increase in LPS in pathogenic proteins, but also promoted the expression of two protective factors in the cardiovascular system and angiogenesis, APJ and VEGF (Figures 8, 9) [31]. Taken together, mangiferin exerted powerful protective effects against LPS-induced neuronal damage and enhanced neuronal survival through anti-neuroinflammation, anti-autophagy, anti-apoptosis, pathogenic protein elimination, and promotion of neurotrophic factor expression. These findings suggest that mangiferin is a potential therapeutic agent against SAE.

## CONCLUSIONS

Mangiferin exhibited neuroprotective effects in an LPS-induced SAE neuronal model through its anti-neuroinflammatory, anti-autophagic, and anti-apoptotic effects.
